# Personalized Care in Late-Stage Parkinson’s Disease: Challenges and Opportunities

**DOI:** 10.3390/jpm12050813

**Published:** 2022-05-18

**Authors:** Margherita Fabbri, Miguel Coelho, Michela Garon, Roberta Biundo, Tiago A. Mestre, Angelo Antonini, on behalf of iCARE-PD Consortium

**Affiliations:** 1 Department of Neurosciences, Clinical Investigation Center CIC 1436, Parkinson Toulouse Expert Centre, NS-Park/FCRIN Network and NeuroToul COEN Center, Toulouse University Hospital, INSERM, University of Toulouse 3, 31062 Toulouse, France; 2 Instituto de Medicina Molecular João Lobo Antunes, 1649-028 Lisboa, Portugal; soarescoelho.miguel@gmail.com; 3 Parkinson and Movement Disorders Unit, Department of Neuroscience, University of Padua, 35131 Padova, Italy; michela.garon@unipd.it (M.G.); angelo.antonini@unipd.it (A.A.); 4 Department of General Psychology, University of Padova, 35131 Padova, Italy; roberta.biundo@unipd.it; 5 Study Center for Neurodegeneration (CESNE), University of Padua, 35131 Padova, Italy; 6 IRCCS San Camillo Hospital, 30126 Venice, Italy; 7 Parkinson’s Disease and Movement Disorders Center, Division of Neurology, Department of Medicine, The Ottawa Hospital Research Institute, University of Ottawa Brain and Mind Institute, Ottawa, ON K1Y 4E9, Canada; tmestre@toh.ca

**Keywords:** Parkinson’s disease, late stage, cognitive impairment, home care, palliative care, caregiver

## Abstract

Late-stage Parkinson’s disease (LSPD) patients are highly dependent on activities of daily living and require significant medical needs. In LSPD, there is a significant caregiver burden and greater health economic impact compared to earlier PD stages. The clinical presentation in LSPD is dominated by motor and non-motor symptoms (NMS) that most of the time have a sub-optimal to no response to dopaminergic treatment, especially when dementia is present. Non-pharmacological interventions, including physiotherapy, cognitive stimulation, speech, occupational therapy, and a specialized PD nurse, assume a key role in LSPD to mitigate the impact of disease milestones or prevent acute clinical worsening and optimize the management of troublesome NMS. However, the feasibility of these approaches is limited by patients’ cognitive impairment and the difficulty in delivering care at home. The present care challenge for LSPD is the ability to offer a person-centered, home-delivered palliative care model based on Advanced Care Planning. An ongoing European multicentric project, PD_Pal, aims to address this challenge.

## 1. Introduction

Parkinson’s disease (PD) evolves throughout different stages, from the early one since the moment of diagnosis to the advanced stage when motor complications appear and become troublesome, up to the late stage (LS), which is the final part of the disease. LSPD has been labeled an “orphan population” due to the little data available on its care needs and the reduced number of available therapeutic options, largely due to the paucity or absence of clinical studies focused on these patients [1,2]. The above-described therapeutic landscape is in sharp contrast with LSPD being the patient group with the greatest impairment and level of dependence, having more complex care needs, and the highest health and economic impact among the different stages of PD stages [3]. In addition, LSPD is undoubtedly expected to have an exponential increase in its prevalence in the next few decades [4].

This narrative review aims to synthesize data on the main unmet care needs and therapeutic challenges in LSPD and puts forward a solution in the form of a personalized care approach. We will review clinical definitions, main care needs, and social burdens in LSPD and provide illustrative clinical care scenarios to frame a proposal for innovative integrated palliative care.

## 2. An Atypical Clinical Phenotype and Unique Clinical Needs

The progression of PD is characterized by a non-linear worsening of motor and non-motor symptoms (NMS) that may be modulated by factors such as age at PD onset, genetic background, predominant motor phenotype, presence of dysautonomia, and REM sleep behavior disorder [5,6]. However, regardless of age at PD onset, disease duration, and the presence of severe motor complications, patients eventually enter the LS, which is clinically homogeneous [1]. Severe dependence in at least half of the activities of daily living (ADLs) and postural instability despite dopaminergic medication (Hoehn and Yahr [HY] 4 or 5) are the defining criteria for LSPD [1,2]. In LSPD, disability is no longer anchored to levodopa-induced motor complications but rather to axial motor symptoms, such as dysphagia, gait impairment, freezing of gait (FoG), postural instability, and NMS, such as hallucinations, cognitive decline, sleep/mood problems, urinary dysfunction, orthostatic hypotension (OH), constipation, and pain. Most of these symptoms have a partial or no response to dopaminergic treatment [1,2,7]. Taken as a whole, the clinical phenotype of LSPD dominated by falls, dysphagia, bilateral more symmetrical Parkinsonian symptoms, and cognitive impairment may evoke the one of atypical Parkinsonism thought after a longer disease course. Among all NMS, cognitive decline and dementia are key contributors to functional decline and loss of independence in ADLs [8]. Indeed, visual hallucinations, falls, and dementia have been identified as the principal disability milestones independent of disease duration and age of PD onset. Together with severe dysphagia and urinary dysfunction, these are associated with increased caregiver burden, a more rapid progression to HY 5, institutionalization, and death [7].

## 3. Therapeutic Challenges: Oral and Non-Pharmacological Approaches

**Figure 1:**
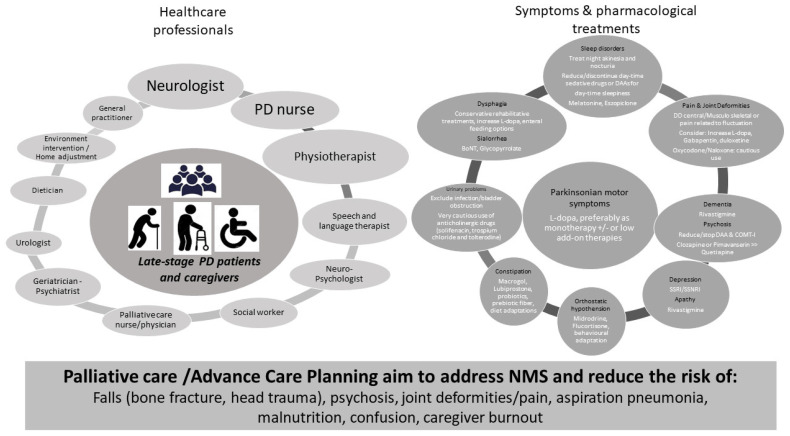
Pillars for Late-stage PD treatment. Left panel: health care professionals involved in LSPD management. Right panel: motor and NMS and available pharmacological options. Bottom: the main objectives of prevention strategies for LSPD patients. COMT−I: Catechol−O-methyl transferase inhibitors; DAAs: dopamine-agonist; SSRI/SNRI: selective serotonin reuptake inhibitor/serotonin and norepinephrine reuptake inhibitor.

The reduction of the risk associated with the above-mentioned disease milestones has a crucial role in the management of LSPD (Figure 1), together with a symptom-based treatment.

Regarding symptomatic treatment, a good practice point consists in using L-dopa, preferentially as monotherapy and at the lowest dose possible. Indeed, other (add-on) dopaminergic therapies, including dopamine-agonists, catechol-O-methyl transferase inhibitors, and monoamine oxidase-B inhibitors, are more likely to induce hallucinations, confusion, or OH among elderly and frail PD patients and, consequently, should be cautiously used in this disease stage [9]. L-dopa has been shown to be effective on rigidity and tremor, especially among non-demented, tremor-dominant patients or those with dyskinesia in LSPD [10,11,12]. These patients may also benefit from cautious L-dopa dose increments for appendicular Parkinsonian symptoms. Conversely, the effect of L-dopa is often modest or barely absent on axial features, which include speech impairment, postural instability, and FoG. Consequently, a disproportionate increment of L-dopa dose to target these features may be unsuccessful and induce significant adverse effects (AEs), namely, worsening confusion or OH.

The management of NMS is based on the application of the clinical evidence available for earlier PD stages [13] and herein summarized in Figure 1 (right panel) [9,14] (see also BOX, Case S1). Nevertheless, the dose therapeutic response, tolerance, and AEs profile may be distinct in LSPD, limiting its applicability. Of note, the regular assessment by a movement disorder specialist, offering specific treatment recommendations, has been shown to have a positive effect on the quality of life (QoL) of LPSD patients, in comparison with the follow-up exclusively by other physicians such as a general practitioner or general neurologist [15].

The implementation of non-pharmacological approaches is an important component in the management of LSPD. Non-pharmacological approaches include physiotherapy for the reduction of risk of falls and joint deformities, speech and language therapy (SLT) for the prevention of aspiration pneumonia, and cognitive training. When considering the complex care needs of PD patients, a multispecialty approach has been suggested as the most suitable solution for tailored and comprehensive care delivery to address care complexity in PD throughout the disease course. Nevertheless, the feasibility of these approaches is yet to be formally evaluated in LSPD, which needs to consider the presence of cognitive impairment and barriers to mobility that lead to an intervention being delivered at home and not in the clinic. A multidisciplinary team ideally involves a wide range of medical specialties and other health care professionals (Figure 1, left panel), implying the development of collaboration strategies amongst team members. There are different levels of care organization which can be coined as multidisciplinary (each discipline is responsible for a specific patient need without standardized coordination), interdisciplinary (a collaboration of the healthcare team members that make group decisions), or integrated care (a care plan involving multiple members of a health care team that is guided by consensus building and engagement of patients as team members). The ideal approach for LSPD patients is that the various components of a care team described above form an integrated palliative care approach to achieve an optimized and effective care delivery (*see paragraph on “Home care”*).

## 4. Management of Device-Aided Therapies in LSPD

LSPD patients previously submitted to device-aided therapies (DAT), i.e., deep brain stimulation (DBS), levodopa-carbidopa intestinal gel (LCIG), or continuous apomorphine subcutaneous infusion (CSAI), represent a small subset of patients in LSPD but are expected to require a more specialized level of care [16,17].

One caveat amongst DATs is CSAI. There is no report of LSPD patients with ongoing CSAI treatment, as likely AEs such as hallucinations, confusion, and OH determine its earlier discontinuation before a patient reaches LSPD. In addition, there are no data about the rate of drop-outs for LSPD in long-term studies of CSAI. Thus, the consideration of this treatment for LSPD patients relies solely on an expert opinion level of evidence. If CSAI is maintained in LSPD, the lowest effective dose should be used (as with any other dopaminergic intervention), likely ranging from 1–3 mg/h over the day, with careful monitorization of AEs, even if no formal recommendation is available. Equally, de novo administration of CSAI should be carefully considered, namely, the risk for poor tolerance (including worsening confusion) to dopamine agonist treatment in LSPD.

Regarding DBS, a common clinical question is the maintenance of this therapy at the time of replacement of the implantable pulse generator in patients no longer having clinically significant motor complications, and for whom the benefit of DBS may be doubtful. A small randomized, double-blind trial attempted to address this question, finding that DBS has a short and long-term benefit, similar to L-dopa, even in LSPD [18]. Worsening of dysphagia and Parkinsonism may occur after switching-off DBS and, at times, after a few days. An algorithm to evaluate the therapeutic benefit of DBS and criteria to discontinue DBS has been proposed [18]. A rule of thumb is to consider increasing neurostimulation with particular caution, especially for the most troublesome axial symptoms, as these likely are a manifestation of disease progression and will not be responsive to dopaminergic treatment [19] and thus to neurostimulation.

Regarding LCIG, there is no study has specifically focused on LSPD. A case-control study has recently shown that elderly PD patients (>80 years old) matched for disease duration and LCIG treatment duration with younger PD patients (<75 years) may have a similar benefit in QoL without a higher occurrence of treatment-related AEs [20]. This study suggests the use of LCIG in elderly patients who still present motor complications and are deemed poor candidates for DBS (see the case in the box). Nevertheless, the indication for LCIG (presence of troublesome motor complications, absence of severe dementia, presence of a caregiver) remains the same and needs to be carefully scrutinized in LSPD. Of note, PEG-J used for LCIG treatment is a viable route for enteral nutrition in LSPD patients with severe dysphagia [21] (see also the box in Case S2).

## 5. Caregiver Burden in LSPD

In PD, the burden of informal caregivers increases with disease progression as patients become more dependent on ADLs [22,23]. A multinational European study found that 588 out of 692 LSPD patients had a primary caregiver [24], who was the spouse or life partner in the majority of the cases. Most LSPD patients lived at home (73.9%) and together with a caregiver in 67% of those cases. Caregivers spent around 7 h per day and 23 days per month assisting or supervising the patient. Only half of the caregivers had assistance from other family members or a professional service. Caregivers reported a high burden measured by the Zarit Burden Interview, and this was similar across the different European countries. Additionally, caregivers self-reported pain/discomfort (58.7%), anxiety/depression (45.5%), and problems with mobility (23.8%), probably reflecting their own advanced age and physical strain [24]. The strongest predictors of caregiver burden were the severity of patient neuropsychiatric symptoms (especially agitation/aggression, apathy/indifference, disinhibition, and irritability/lability) and other NMS (attention/memory and mood/cognition), male gender and home as the living setting. Despite the significant burden, caregivers expressed a sense of loyalty and responsibility to the patient and partner, a sense of belonging together, and that caring well for the patient delayed institutionalization [25], though the increased strain on caregivers was a strong predictor of nursing home placement of patients. Indeed, caregivers had mixed feelings about nursing home placement as they also accepted the need to move to a nursing home once living at home was not feasible or safe for the patient [25]. Caregivers also reported feeling “guilty, if not there” for the patients and were particularly concerned about the occurrence of falls [25,26]. The QoL of caregivers of LSPD patients was found to be severely impaired, and the cognition impairment of patients and caring for a male patient [26] were the strongest predictors of poorer QoL.

## 6. Management of LSPD Living in Nursing Homes

In the USA, 6.8% of nursing home residents have a diagnosis of Parkinsonism [27]. On the other hand, about one-third of LSPD patients live in nursing homes [28], although prevalence varies between countries. LSPD patients living in nursing homes were found to be slightly older, have worse motor disability and ADL scores, and spend more time in the *off* period but have fewer and less disabling dyskinesia [28]. One study found that 44% of LSPD patients in nursing homes were in the *off state* for most of the time, 25% were treated with less than 400 mg/day of L-dopa, and 8% were not taking L-dopa at all [29]. Interestingly, patients had fewer falls despite higher motor disability in items related to standing and walking compared to patients living at home. Additionally, patients living in nursing homes were more likely to have dementia and a higher burden of NMS, namely neuropsychiatric symptoms such as delusions, hallucinations, and depression. Despite a more severe disease, LSPD patients in nursing homes have a similar sense of well-being and satisfaction with care compared to those living at home [28]. These differences in clinical characteristics of patients living in nursing homes have practical implications for management, although we must keep in mind that some of the observed clinical differences, such as the prevalence of falls, may reflect different care being delivered at nursing homes. One study found that only one-third of PD patients living in long-term care facilities in North America had outpatient neurology care, suggesting that appropriate care varies between nursing homes and homes [30]. Nevertheless, this may vary between healthcare systems, as a multicenter European study found no difference in the levodopa equivalent daily dose between LSPD patients living in nursing homes and those living in their own homes [28]. In this study, those living in nursing homes took less frequent dopamine agonists, but anxiolytics, hypnotics, antipsychotics, and antidepressants were prescribed more often. The results of a randomized controlled trial in 91 LSPD patients (60% in nursing homes) suggest that the implementation of PD experts’ recommendations by treating physicians regarding medication and allied health interventions was associated with improved motor and nonmotor disability and better QoL in LSPD patients [15]. Simple measures such as addressing the knowledge of staff at nursing homes on PD-related issues using educational programs have been found to significantly improve patients’ motor symptoms (UPDRS-III) and QoL up to 12 months [31]. In fact, patients and caregivers feel that knowledge about PD is fundamental to experiencing good care, and the potential lack of knowledge about PD care in residential staff is a major barrier to moving into nursing homes [25].

## 7. Risks and Management of LSPD during Hospitalizations Due to Systemic Illness

There are no specific data regarding the hospitalization of LSPD patients. The rate of hospital admissions of PD patients seems to increase with a longer time from diagnosis, suggesting that hospitalizations are higher in more disabled PD patients [32]. Longitudinal data found that 39% of 9998 PD patients were hospitalized over a follow-up of 5.1 years [32], and about 80% of hospital admissions were non-elective [33,34]. The most common reasons for hospital admission were consistent across studies and included more frequent infections, namely pneumonia and urinary tract infection, falls and bone fractures, motor deterioration, neuropsychiatric symptoms, and gastrointestinal dysfunction such as dysphagia, constipation, and vomiting [32,33,34,35,36,37]. PD patients stayed 2–14 days longer in hospital than non-PD patients [34,38], and this was particularly true in those with dementia [39]. In-hospital mortality was between 3.9–10% [33,34,37], a higher rate compared to non-PD patients [34], and one study found that two-thirds of those dying in the hospital were in HY stage 4 or 5 [40]. After hospital discharge, there was an increased risk of patients requiring nursing home placement [36].

During a hospital or emergency department admission, several issues may emerge. One study found that the PD medication was stopped, omitted, or prescribed inappropriately in 74% of the cases during hospital admissions and that non-compliance to the medication schedule was common [41]. Antidopaminergic medication was prescribed in 11% of the cases, which was associated with an increased risk of falls. During the hospital admission, urinary tract infections, *delirium*, and pressure ulcers were common acute events, particularly in the perioperative period after a patient with PD had surgery [38]. In fact, a survey found that many PD expert centers are concerned about the quality of PD-specific care provided during hospitalization, namely, the adherence to a PD medication schedule and the knowledge about contra-indicated medications for PD patients by care teams [42]. 

## 8. Home Care for Late-Stage PD

In most of the world, PD outpatient care is currently fragmented, institution-based, and shows insufficient multispecialty collaboration between health care providers, with unmet care needs in this population [43]. Despite some attempts with clinical trials embracing a home care delivery model instead of institutionalized care, LSPD patients experience problems in coordination and continuity of care as the progressive loss of mobility, and cognitive decline limit their ability to physically reach expert care [44]. Indeed, the evaluation and implementation of non-pharmacological interventions (e.g., physiotherapy, cognitive stimulation, speech and occupational therapies, and specialized PD nurses) are limited by the presence of significant cognitive deficits [45] and the difficulty in conducting studies at home [8,46]. Provided the lack of institutional support for home care, patients rely on their caregivers, who lack preparation for this role and experience most of the financial and psychological burden of the disease. Interestingly, the lack of expertise in PD of health care professionals providing home care [44] contributes to the increased burden experienced by caregivers. A new and holistic patient-centered model of care is therefore warranted. In LSPD, the adoption of a palliative care symptom-oriented paradigm could be considered, especially if delivered at home, with potential benefits in terms of reduced caregiver burden and improvement in QoL. Although traditionally associated with oncological illnesses, palliative care has been recently applied to chronic diseases beyond end-of-life care [43]. In fact, it is advocated for this approach to begin earlier in neurodegenerative disorders, where a cure is not possible, and functional decline is complicated by unpredictable comorbidities and frailties. In this clinical context, palliative care should be seen as Advance Care Planning (ACP), an approach aimed at the patient’s family unit, with the purpose of relieving them from suffering and addressing medical symptoms and psychosocial and spiritual needs [47]. The definition of ACP emphasizes the need for a multidisciplinary team, integrating diverse aspects of care to enhance patients’ and their families’ QoL and to assist them in expressing their wishes about symptoms and crisis management. Nevertheless, several barriers prevent neurologists from adopting palliative care. First, general physicians lack fundamental palliative care skills and education, and there are no clear referral guidelines for palliative care or ACP. Second, patients are often reluctant to adhere to palliative care or ACP due to the misconception that it is the same as end-of-life care, and clinicians are concerned about diminishing their hope when proposing these approaches [48]. To address these challenges, the integration of a palliative care model focused on patients’ QoL along the course of the disease is being evaluated in the context of a multicentric project in Europe (PD_Pal, https://www.pdpal.eu, accessed on 17 May 2022). The project includes an ongoing multicenter, open-label randomized controlled trial with a parallel group design (clinical trial registration n NL8180). The PD_Pal model of palliative care comprises hospice care through the development of guidelines for palliative care focused on end-of-life care, an outpatient palliative care regimen with regular visits, continuous review of the care plan, and active participation of family members to preserve the patient’s QoL. This model of care is based on a multidisciplinary approach: different health care professionals (movement disorders specialist, palliative care physician, psychologist, psychiatrist, physiotherapist, and palliative care nurse) provide comprehensive assessments, having at the center of the integrated, proactive palliative care plan a trained PD nurse. The “PD_Pal intervention” focuses on patients and caregivers as follows: trained nurses provide support for care coordination, make referrals to specialists and initiate ACP conversations. Indeed, one of the outcomes in PD_Pal is the percentage of patients with documented ACP decisions at a 6-month follow-up. Secondary endpoints include caregivers’ and patients’ QoL, perceived care coordination, symptoms management, and reduced patient symptom burden as well as cost-effectiveness [49]. The ultimate goal of the project is to provide a model that is feasible, cost-effective, and can inform new guidelines about the timing and referral for ACP. The sustainability of a palliative care approach has been suggested in previous studies showing a reduction in the costs of hospital care secondary to the implementation of a community-based specialist palliative care across multiple life-threatening conditions [50]. The PD_Pal model is also evaluating the implementation of home monitoring through telemedicine and wearable devices for the assessment of motor symptoms and as an enabler of the PD_Pal intervention [51]. Telemedicine is hypothesized to have greater utility for LSPD patients living at home by potentially delaying advanced disease complications and enabling more continuous care delivery [50,52].

Moreover, to educate healthcare professionals, patients, and caregivers, the PD_Pal project developed the “Best Care for People with Late-Stage Parkinson’s Disease” curriculum toolkit implemented as a Massive Online Open Course (MOOC, www.pdpal.eu/mooc, accessed on 24 April 2022) for ACP training [53].

## 9. Conclusions

The prevalence of LSPD patients will increase based on the expected exponential increase in PD prevalence in the general population [4], forcing physicians, other health care professionals, and health care systems to deal with a PD population in need of specialized and complex care, ideally delivered in a coordinated framework.

In this review, we covered the clinical definition of LSPD, highlighting its main bothersome motor and NMS and providing evidence on how those symptoms can negatively affect caregivers. We covered aspects of frailty that impact these patients once they are admitted into hospitals or nursing homes. Presently, we can identify LSPD patients more likely to respond to dopaminergic treatments and those at higher risk for worse outcomes who thus require a closer follow-up. Despite this knowledge, the management of axial motor symptoms and NMS remains challenging due to the reduced number of pharmacological options available, care fragmentation, and reduced feasibility of non-pharmacological approaches in the presence of a significant and progressive cognitive burden. We propose a feasible integrated model of care that could reach patients at home, offering a real-life community-based specialist palliative care. With these concepts in mind, the PD_Pal project has been developed, hoping to provide robust evidence on the effectiveness of ACP implementation in LSPD as well as a positive effect on patients’ and caregivers’ QoL, in addition to the impact of home monitoring by wearable sensors and a healthcare education program focused on ACP.

## Supplementary Materials

The following supporting information can be downloaded at: https://www.mdpi.com/article/10.3390/jpm12050813/s1. Box with case study. Case S1: Late-onset PD patient with psychosis and weight loss; Case S2: Early onset PD patient with GBA mutation and challenging management of motor fluctuations.
