# Supplementary material for: Personalized Care in Late-Stage Parkinson’s Disease: Challenges and Opportunities
Source: J Pers Med. 2022 May 18;12(5):813. doi: 10.3390/jpm12050813 (PMC9147917; doi:10.3390/jpm12050813)
Supplement: Supplementary file 1 [file jpm-12-00813-s001.zip › jpm-1719876-supplementary.pdf]

Supplementary material

BOX – Case studies for clinical challenges in late-stage PD

Case S1. Late-onset PD patient with psychosis and weight loss.

Mr M was diagnosed at the age of 69 with an akinetic-rigid idiopathic PD. He had an initial good response to L-dopa/carbidopa treatment (400 mg/day). A low daily dose of ropinirole 4 mg was not well tolerated due to excessive daytime sleepiness. At the age of 74, he switched to L-dopa/carbidopa/entacapone (200 mg q.i.d.) due to the presence of a troublesome predictable wearing-off, despite 5 daily doses of L-dopa. No cognitive complains were reported by the patient and his wife at that time. Two years later, his wife suddenly deceased. At the age of 78, he came at the outpatient regular visit, with his son, referring decline in cognitive performances, persistent visual hallucinations, more severe at night, several falls each week and weight loss (8 kg over 12 months). On exam, he would fall in the pull test, he had a FoG while turning, rigidity was mild and bradykinesia was moderate when ON Medication (HY 4). Persistent sadness and loss of initiative were endorsed by the patient and his son, in addition to reduced food intake. Motor fluctuations were not reported. All L-dopa/carbidopa/entacapone intakes were switched into L-dopa/carbidopa at the same dose (1000mg/day), which lead to a remission of hallucinations. He had mild dysphagia, but he had only a meal daily, which was attributed to loss of initiative and increasing difficulties in ADLs. A speech and language therapy assessment was performed and behavioural adaptation during meals implemented along with home meal preparation assistance by the social worker. At the age of 80, rivastigmine treatment was implemented due to cognitive impairment, though not tolerated due to gastrointestinal AEs. A treatment with clozapine 12.5 mg in the evening was started to manage persistent night-time hallucinations. L-dopa/carbidopa was maintained at a reduced dose (800mg/day).

Case S2. Early onset PD patient with GBA mutation and challenging management of motor fluctuations.

Ms R was diagnosed at the age of 47 with young-onset akinetic-rigid PD, with optimal response to a dopamine agonist (pramipexole 2.1 mg/day) but early need for L-dopa/carbidopa w, due worsening motor symptoms (rapidly up to 400 mg/day). She was screened positive for a heterozygous mutation (L444P, c.1448T>C) in the GBA gene. At the age of 50, pramipexole dose was reduced at 0.52 mg/day due to impulsive control disorders (gambling) and rare visual hallucinations, which ceased after its dose reduction. At the age of 56 years, due to severe motor fluctuations he was evaluated for a possible device-aided therapy. Acute levodopa challenge test showed a good L-dopa responsiveness, but with significant axial signs post-challenge (HY 4 in Med ON and HY 5 in Med Off, mild FoG and postural instability). Neuropsychological assessment disclosed mild cognitive impairment (MCI). Treatment with LCIG was implemented, due to the persistence of severe motor fluctuations and deep brain stimulation contraindications (axial signs and MCI). LCIG allowed an improvement of 50% of wearing-off duration and mild improvement

of gait, even in the presence of FoG and postural instability. At the age of 65, now with 18 years of disease duration and 7 years of LCIG treatment, a nocturnal enteral feeding by second PEG lumen was started due to a severe dysphagia, malnutrition signs, a hospitalisation for aspiration pneumoniae and dyskinesia increment. Gait was possible with a walker only for few meters. LCIG dose was reduced (from 5 cc/h to 4.2 cc/h, from 7 am to 11 pm). A daytime feeding pleasure was maintained, the enteral feeding and reduction of dyskinesia with LCIG allowed for significant weight gain.
